# Variety and quantity of dietary protein intake from different sources and risk of new-onset diabetes: a Nationwide Cohort Study in China

**DOI:** 10.1186/s12916-021-02199-8

**Published:** 2022-01-13

**Authors:** Chun Zhou, Chengzhang Liu, Zhuxian Zhang, Mengyi Liu, Yuanyuan Zhang, Huan Li, Panpan He, Qinqin Li, Xianhui Qin

**Affiliations:** 1 National Clinical Research Center for Kidney Disease, State Key Laboratory for Organ Failure Research, Guangdong Provincial Key Laboratory of Renal Failure Research, Guangzhou Regenerative Medicine and Health Guangdong Laboratory, Division of Nephrology, Nanfang Hospital, Southern Medical University, Guangzhou, 510515 China; 2 Department of Epidemiology and Biostatistics, School of Public Health, Anhui Medical University, Hefei, 230032 China; 3 Institute of Biomedicine, Anhui Medical University, Hefei, 230032 China

**Keywords:** Dietary protein intake, Variety, Quantity, different food sources, New-onset diabetes

## Abstract

**Background:**

The relation of the variety and quantity of different sources of dietary proteins intake and diabetes remains uncertain. We aimed to investigate the associations between the variety and quantity of proteins intake from eight major food sources and new-onset diabetes, using data from the China Health and Nutrition Survey (CHNS).

**Methods:**

16,260 participants without diabetes at baseline from CHNS were included. Dietary intake was measured by three consecutive 24-h dietary recalls combined with a household food inventory. The variety score of protein sources was defined as the number of protein sources consumed at the appropriate level, accounting for both types and quantity of proteins. New-onset diabetes was defined as self-reported physician-diagnosed diabetes or fasting glucose ≥7.0mmol/L or glycated hemoglobin ≥6.5% during the follow-up.

**Results:**

During a median follow-up of 9.0 years, 1100 (6.8%) subjects developed diabetes. Overall, there were U-shaped associations of percentages energy from total protein, whole grain-derived and poultry-derived proteins with new-onset diabetes; J-shaped associations of unprocessed or processed red meat-derived proteins with new-onset diabetes; a reverse J-shaped association of the fish-derived protein with new-onset diabetes; L-shaped associations of egg-derived and legume-derived proteins with new-onset diabetes; and a reverse L-shaped association of the refined grain-derived protein with new-onset diabetes (all *P* values for nonlinearity<0.001). Moreover, a significantly lower risk of new-onset diabetes was found in those with a higher variety score of protein sources (per score increment; HR, 0.69; 95%CI, 0.65–0.72).

**Conclusions:**

There was an inverse association between the variety of proteins with appropriate quantity from different food sources and new-onset diabetes.

**Supplementary Information:**

The online version contains supplementary material available at 10.1186/s12916-021-02199-8.

## Background

A healthy diet has been revealed to play a crucial role in type 2 diabetes prevention and management [1]. Numerous studies have focused on the impact of macronutrients on diabetes risk [1–3]. Some previous studies suggested that protein/amino acids may possibly have some effects on insulin release, thereby affecting the clearance of glucose from the blood [4]. It has been reported that protein intake is a rather heterogeneous exposure and proteins from specific food sources may differentially affect health outcomes [5]. However, the role of the variety of protein sources, and the quantity of specific sources of dietary proteins intake on diabetes risk have not been explored comprehensively in previous studies.

Recent meta-analyses of prospective cohorts reported that high total protein and animal protein intakes were associated with an increased risk of diabetes, whereas evidence for plant protein was mixed [6–9]. Moreover, most of the previous studies have investigated relations of intake of total protein, total animal protein, and total plant protein with diabetes risk. Few studies [10, 11] have been conducted to examine the associations between protein intake from more specific food sources, especially plant protein sources, for which the current evidence is very inconsistent, and new-onset diabetes. More importantly, few previous studies have been conducted using the dietary protein intake data continuously, which may allow for the possibility of a non-linear association between protein intake and the risk of diabetes and provide more detailed information. Furthermore, although some studies have suggested that a greater variety of dairy, fruit, and vegetable appeared important for a reduced risk of diabetes [12, 13], to date, the relation of the variety of protein sources with new-onset diabetes has not yet been examined.

To address the important knowledge gaps, our study aimed to investigate the prospective associations between the variety and quantity of proteins intake from different food sources (whole and refined grain, processed and unprocessed red meat, poultry, fish, egg, and legumes) and new-onset diabetes in general Chinese population, using data from China Health and Nutrition Survey (CHNS).

## Methods

### Study design and participants

Details of the study design and some major results of the CHNS have been described elsewhere [14–18]. Briefly, CHNS is an ongoing, national, multipurpose, longitudinal, open cohort study initiated in 1989 and has been followed up every 2–4 years. The CHNS rounds have been completed in 1989, 1991, 1993, 1997, 2000, 2004, 2006, 2009, 2011, and 2015. The response rates had been estimated as about 80% from 1989 to 2006 at an individual level [14]. By 2011, the provinces included in the CHNS constituted 47% of China’s population [15].

The present study was based on 7 rounds of CHNS data from 1997 to 2015 (1997, 2000, 2004, 2006, 2009, 2011, and 2015), including a total of 94,532 person-waves (*n*=32,572). We first excluded participants who were pregnant or <18 years old. Among the remaining participants (*n*=25,960; including 76,500 person-waves), those with missing diabetes diagnosis (*n*=146; including 1034 person-waves) or with only one survey wave (*n*=8919; including 8919 person-waves) were further excluded. Therefore, a cohort based on 16,895 participants (66,547 person-waves) with two or more survey waves was identified, and the first survey round was considered as baseline. The characteristics of the included (*n*=16895; including 66,547 person-waves) and excluded (*n*=9065; including 9,953 person-waves) population were shown in Additional file 1: Table S1. Of the 16,895 participants, 444 participants with diabetes (fasting plasma glucose ≥7.0 mmol/L or glycated hemoglobin (HbA1c) ≥6.5% or previously diagnosed by a physician) at baseline, 103 with missing dietary protein data, and 88 with extreme dietary energy data (male: >4200 or <800 kcal/day; female: >3600 or <600 kcal/day) [19] were further excluded. Finally, a total of 16,260 participants were included in the final analysis (Additional file 1: Fig. S1).

The institutional review boards of the University of North Carolina at Chapel Hill and the National Institute of Nutrition and Food Safety, and the Chinese Center for Disease Control and Prevention, approved the study. Each participant provided their written informed consent. The data and study materials that support the findings of this study can be found from the CHNS official website (http://www.cpc.unc.edu/projects/china).

### Dietary nutrient intakes

Dietary measurements in CHNS are described in detail elsewhere [20]. Briefly, both individual and household level data were collected in each survey round. Dietary information was collected by 3-day dietary recalls in combination with using a 3-day food-weighted method to assess cooking oil and condiment consumption. The 3 consecutive days were randomly allocated from Monday to Sunday and are almost equally balanced across the seven days of the week for each sampling unit. Nutrient intakes were calculated using the China food composition tables (FCTs) [21–23]. The accuracy of 24-h dietary recall designed to assess energy and nutrient intake has been validated [20].

In the analyses, 3-day average intakes of dietary macronutrients and micronutrients in each round were calculated. Repeated 3-day dietary recalls may reduce the day-to-day variation of dietary intake and collect more complete food information. Moreover, all values of each nutrient in the analyses, if not specified, were presented as the cumulative averages, using all results from baseline to the last visit prior to the date of new-onset diabetes, or using all results among participants without new-onset diabetes, to represent long-term dietary intake status and minimize within-person variation.

Furthermore, in the present study, total protein was divided into specific sourced proteins. Food sources constituting these subtypes are shown in Additional file 1: Table S2 [24, 25]. Of those, whole and refined grain, processed and unprocessed red meat, poultry, fish, egg, and legumes were the 8 major sources of proteins in this population.

The variety score of protein sources was calculated as the sum of total numbers of the 8 major food sources of proteins consumed in the appropriate quantities during the study period. The appropriate quantity for each major food source of protein means a window of consumption level (% of energy, shown in the results section) where the risk of new-onset diabetes is relatively lowest. In other words, if participants consumed one of the 8 major food sources of protein at an appropriate quantity during the entire study period, they will get one point, with a maximal score of 8. The variety score of protein sources may account for both types and quantity of proteins intake.

### Assessment of blood pressure and other covariates

After the participants had rested for 5 min, seated blood pressure (BP) was measured by trained research staff using a mercury manometer, following the standard method. Triplicate measurements on the same arm were taken in a quiet and bright room. The mean systolic blood pressure (SBP) and diastolic blood pressure (DBP) of the three independent measures were used in the analysis.

Information on age, sex, urban or rural residents, region, education level, occupation, physical activity, smoking, and drinking status was obtained from the questionnaires at each follow-up survey. Height and weight were measured following a standard procedure with calibrated equipment. Body mass index (BMI) was calculated as weight (kg) by height squared (m^2^). The level of physical activity was the product of the self-reported time spent in each activity multiplied by specific metabolic equivalent (MET) values [26]. For all the nondietary covariates, we used the baseline year measurements [27].

### Study outcome

Diabetes status was identified by the questionnaire-based interview at each follow-up. Answering “yes” to the question “has a doctor ever told you that you suffer from diabetes?” was defined as having self-reported physician-diagnosed diabetes [28]. In addition, overnight-fasting blood samples were collected and assayed only in 2009. Therefore, an additional criterion (fasting blood glucose ≥ 7.0 mmol/L or HbA1c ≥ 6.5%) [29, 30] was added for outcome ascertainment in 2009. We have examined the concordance between questionnaire-based diagnosis and HbA1c/glycemia-based diagnosis among the 8202 participants in the 2009 wave. Among the 247 participants with self-reported physician-diagnosed diabetes in the 2009 wave, 182 (74%) were diagnosed with fasting glucose ≥ 7.0 mmol/L or HbA1c ≥ 6.5%. Of the remaining 65 participants, 58 were under glucose-lowering treatment which may maintain fasting glucose and HbA1c in a relatively normal level.

### Statistical analysis

Population characteristics are presented as mean ± standard deviations (SDs) for continuous variables and proportions for categorical variables. Differences in population characteristics by dietary total protein intake quintiles (% of energy, <10.6, 10.6 ≤11.6, 11.6 ≤12.6, 12.6 ≤14.0, ≥14.0) were compared using ANOVA tests, Kruskal-Wallis test, or chi-square tests, accordingly.

The year of each participant’s first entry into the survey was considered as a baseline. The follow-up person-time for each participant was calculated from baseline until a first new-onset diabetes diagnosis (the middle date between the survey of the first diagnosis and the nearest survey before), the last survey round before the participant’s departure from the survey, or the end of the latest survey (2015), whichever came first. Participants were censored on the date of the last survey round before the participant’s departure from the survey, or the end of the latest survey. Incidence rates of new-onset diabetes, expressed as person-years, were calculated as the sum of follow-up years for participants.

Variables that are known to be traditional or suspected risk factors for diabetes or variables that showed significant differences among different protein levels were chosen as the covariates in the adjusted models. The relations of energy from total protein, proteins from different food sources (whole and refined grain, processed and unprocessed red meat, poultry, fish, egg, and legumes) with new-onset diabetes were estimated using Cox proportional hazards models. We built the isocaloric models to estimate the relative risk of new-onset diabetes. The rationale is that in the isocaloric models, the reduction of one macronutrient as a percent of total energy intake will be replaced by the same proportion of energy from another macronutrient. Model 1 included the adjustments with age, sex, body mass index (BMI), occupations at baseline, and cumulative average total energy intake. Model 2 included the adjustments in model 1 plus education level, region, smoking status, SBP, DBP, urban or rural residents, and physical activity (low, moderate, high) at baseline, as well as cumulative average fiber intake, sodium to potassium intake ratio and fat intake (% of energy). Moreover, mutual adjustments for a cumulative average intake of other sources of dietary proteins (% of energy) were further included for the association between proteins from different food sources and new-onset diabetes. To test the proportional hazards assumption, the significance of the interaction between exposures and log-transformed follow-up time was assessed, and no clear evidence of violation was detected. We also used restricted cubic splines (RCS) with 4 knots (20%, 40%, 60%, 80% of proteins intake) to express the potentially non-linear relationship of variety score of protein sources, energy from total protein and proteins from different food sources (whole and refined grain, processed and unprocessed red meat, poultry, fish, egg, and legumes) with new-onset diabetes with adjustments in model 2.

The appropriate quantity for each major food source of protein was determined by assessing different sources of proteins intake (% of energy) as categorical variables (quartiles or quintiles), and choosing the corresponding protein categories with the relatively lowest risk of new-onset diabetes. For each protein whose proportion of non-consumers was less than 20%, participants were divided into five groups according to quintiles of the protein intake, and quintile 1 was used as the reference. For each protein whose proportion of non-consumers was over 20%, consumers were divided into four groups according to quartiles and non-consumers were used as the reference. Moreover, possible modifications of the association between total protein intake, variety score of protein source, and new-onset diabetes were evaluated by stratified analyses and interaction testing.

There were missing values on BMI (*n*= 1522), smoking status (*n*= 49), SBP (*n*= 1476), DBP (*n*= 1477), education level (*n*= 344), occupations (*n*=152), and physical activity (*n*=180) at baseline, and those with missing values of covariates were excluded in the main analysis. Furthermore, multiple imputations were used to handle missing covariates in the sensitivity analysis.

We consider a two-sided *P* value<0.05 as statistically significant in all analyses. All statistical analyses were conducted using R version 3.6.1.

## Results

### Population characteristics of study participants

As demonstrated in Additional file 1: Fig. S1, the final analytic cohort included a total of 16,260 participants. The mean percentage of energy intake from total protein was 12.4% (SD: 2.4), and the mean level of variety score of protein sources was 3.5 (SD: 1.5). Grains followed by red meat, legumes, fish, egg, and poultry are the major sources of protein, accounting for more than 80% of dietary protein intake (Additional file 1: Table S2). In addition, we performed the longitudinal comparisons of macronutrients intake from 1997 to 2011 (Additional file 1: Fig. S2) and found that the median level of the percent of energy intake from total protein was maintained around 12% over the 15 years.

Population characteristics for the study participants by total protein intake quintiles were illustrated in Table 1. The mean age of the participants was 43.1±15.3 years, and 51.2% were females. Participants with higher total dietary protein intake were more likely to be males, alcohol drinkers, and unemployed and less likely to be farmers; had higher SBP, DBP, BMI, and education levels, higher intake of fiber, lower intake of carbohydrate, and lower sodium to potassium intake ratio and physically active levels.

**Table 1:** Population characteristics by quintiles of energy from cumulative average total protein intake (% of energy)

Characteristics	Quintiles of cumulative average protein intake, % of energy					*P* value
	Q1 (<10.6)	Q2 (10.6 ≤ 11.6)	Q3 (11.6 ≤ 12.6)	Q4 (12.6 ≤ 14.0)	Q5 (≥14.0)	
*N*	3252	3252	3252	3252	3252	
Male, No. (%)	1428 (43.9)	1549 (47.6)	1613 (49.6)	1691 (52.0)	1660 (51.0)	< 0.001
Age, years	44.0±16.0	42.3±14.8	42.3±14.7	42.5±15.5	44.6±15.4	< 0.001
Systolic blood pressure, mmHg	119.5±19.1	118.6±16.6	119.0±17.1	119.9±17.2	121.6 ±16.2	< 0.001
Diastolic blood pressure, mmHg	77.0±11.4	77.0±10.7	77.3±10.6	77.6±10.7	78.6±10.1	< 0.001
Body mass index, kg/m^2^	22.2±3.3	22.5±3.3)	22.7±3.2	23.1±3.3	23.6±3.4	< 0.001
Smoking, No. (%)	965 (29.8)	1027 (31.7)	1031 (31.8)	1019 (31.5)	1049 (32.3)	0.232
Alcohol drinking, No. (%)	1000 (31.2)	1078 (33.6)	1131 (35.1)	1224 (38.1)	1306 (40.4)	< 0.001
Urban residents, No. (%)	719 (22.1)	894 (27.5)	1069 (32.9)	1455 (44.7)	2004 (61.6)	< 0.001
Regions, No. (%)						< 0.001
Central	1306 (40.2)	1357 (41.7)	1604 (49.3)	1682 (51.7)	1870 (57.5)	
North	688 (21.2)	735 (22.6)	620 (19.1)	621 (19.1)	820 (25.2)	
South	1258 (38.7)	1160 (35.7)	1028 (31.6)	949 (29.2)	562 (17.3)	
Physical activity, No. (%)						< 0.001
Low	962 (29.8)	982 (30.6)	1077 (33.6)	1150 (35.7)	1195 (37.1)	
Moderate	871 (27.0)	959 (29.9)	1017 (31.8)	1183 (36.8)	1326 (41.1)	
High	1391 (43.1)	1269 (39.5)	1109 (34.6)	885 (27.5)	704 (21.8)	
Occupation, No. (%)						< 0.001
Farmer	1625 (50.6)	1488 (46.3)	1252 (38.9)	738 (22.9)	225 (7.0)	
Worker	250 (7.8)	311 (9.7)	411 (12.8)	481 (14.9)	442 (13.7)	
Unemployed	786 (24.5)	782 (24.3)	828 (25.7)	974 (30.2)	1218 (37.6)	
Other	549 (17.1)	631 (19.6)	731 (22.7)	1035 (32.1)	1351 (41.7)	
Education, No. (%)						< 0.001
Illiteracy	993 (31.3)	725 (22.9)	685 (21.5)	510 (16.1)	324 (10.1)	
Primary school	770 (24.3)	790 (24.9)	679 (21.3)	490 (15.4)	369 (11.5)	
Middle school	929 (29.3)	1017 (32.1)	1092 (34.3)	1093 (34.4)	962 (29.9)	
High school or above	478 (15.1)	638 (20.1)	728 (22.9)	1084 (34.1)	1560 (48.5)	
**Cumulative average dietary intake**						
Variety of protein	3.0±1.5	3.4±1.5	3.6±1.5	3.8±1.4	3.6±1.4	< 0.001
Energy, Kcal/day	2200.9±539.6	2201.4±479.7	2181.8±470.9	2138.5±498.6	1964.3±520.0	< 0.001
Fat, % of energy	31.8±11.6	29.8±9.1	29.9±9.3	32.1±9.0	34.5±8.8	< 0.001
Carbohydrate, % of energy	58.5±11.4	59.1±9.2	58.0±9.3	54.7±9.1	49.5±9.2	< 0.001
Protein, g/day	53.5±14.2	61.3±13.4	65.9±14.3	70.6±16.6	78.4±22.3	< 0.001
Fiber, g/d	9.8±5.1	10.8±5.0	11.2±5.3	10.8±5.7	10.2±6.5	< 0.001
Sodium to potassium ratio	3.9±2.6	3.3±1.8	3.2±1.8	3.1±1.7	2.9±2.1	< 0.001

Variables are presented as mean ± SDs or *N* (%)

### Relations of dietary total protein and proteins from different food sources (% of energy) with new-onset diabetes

Of the included 16,260 participants, during a median follow-up duration of 9.0 years (interquartile range: 4.1 to 15.1 years), 1100 participants (6.8%) developed new-onset diabetes, and 937 participants died.

**Fig. 1:**
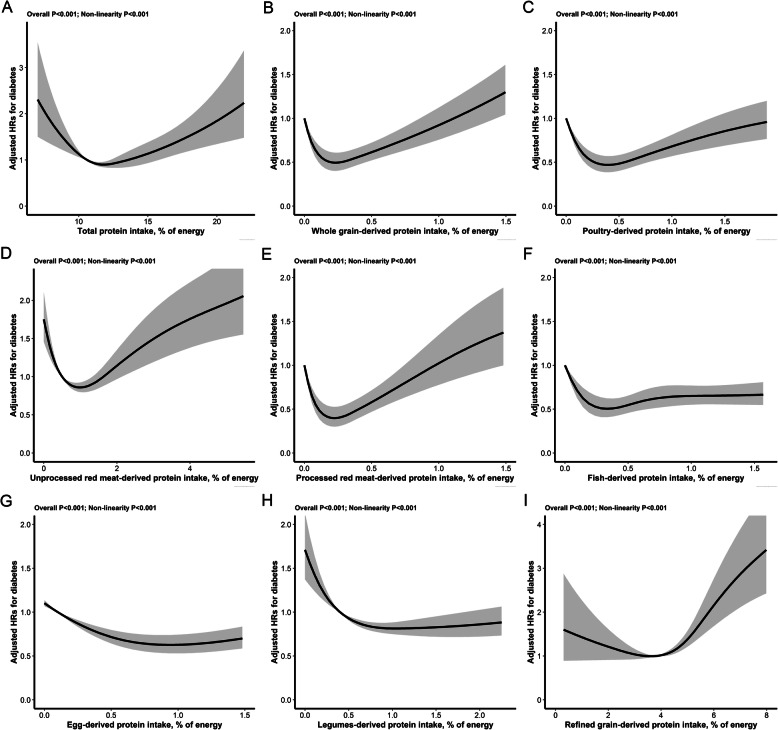
The relations of specific sourced protein intake (% of energy) with new-onset diabetes^*^_._^*^Adjusted for age, sex, BMI, occupations, education level, region, smoking status, SBP, DBP, urban or rural residents, physical activity (low, moderate, high) at baseline, as well as cumulative average, total energy intake, fiber intake, sodium to potassium intake ratio, and fat intake, in addition to mutual adjustments for other specific dietary protein source (% energy). (**A** total protein intake, **B** whole grain-derived protein, **C** poultry-derived protein, **D** unprocessed-derived protein, **E** processed-derived protein, **F** fish-derived protein, **G** egg-derived protein, **H** legume-derived protein, **I** refined grain-derived protein)

Overall, there was a U-shaped association between the percentage energy from dietary total protein intake and new-onset diabetes (*P* for nonlinearity <0.001). Accordingly, when the percentage energy from total protein intake was assessed in quintiles, significantly higher risks of new-onset diabetes were found in participants in the first quintile (<10.6% of energy from total protein, adjusted HR, 1.27; 95%CI 1.07, 1.50) and the fifth quintile (≥14.0% of energy from total protein, adjusted HR, 1.35; 95%CI 1.13, 1.60), compared with those in the 2–4 quintiles (10.6% ≤ 14.0% of energy from total protein) (Fig. 1A, Table 2). Similar results were found with the exclusion of early new-onset diabetes that occurred within the first 2 years after enrollment, using multiple imputations to handle missing values at baseline, or further adjusting for dietary cholesterol intake (Additional file 1: Table S3). In addition, 937 participants died during the follow-up period and accounting for the competing risk of death also did not substantially change the results (Additional file 1: Table S3).

**Table 2:** The relation of total protein intake (% of energy) with new-onset diabetes

Protein, % of energy	No. of case	Person-years	Model 1	*P* value	Model 2	*P* value
			HR (95%CI)		HR (95%CI)	
Quintiles	1100					
Q1 (<10.6)	227	32885	1.20 (0.99, 1.45)	0.07	1.20 (0.99, 1.47)	0.07
Q2 (10.6 ≤ 11.6)	229	36006	*Ref*		*Ref*	
Q3 (11.6 ≤ 12.6)	223	35790	0.91 (0.75, 1.11)	0.371	0.91 (0.75, 1.12)	0.381
Q4 (12.6 ≤ 14.0)	208	31776	0.94 (0.77, 1.15)	0.528	0.91 (0.74, 1.12)	0.385
Q5(≥14.0)	213	22473	1.30 (1.06, 1.60)	0.011	1.30 (1.05, 1.62)	0.015
Categories						
Q1 (<10.6)	227	32885	1.26 (1.07, 1.48)	0.005	1.27 (1.07, 1.51)	0.005
Q2–4 (10.6 ≤ 14.0)	660	103572	*ref*		*ref*	
Q5 (≥14.0)	213	22473	1.38 (1.16, 1.63)	<0.001	1.39 (1.17, 1.66)	<0.001

Model 1: Adjusted for age, sex, BMI, and occupations at baseline, and cumulative average total energy intake

Model 2: Adjusted for variables in model 1 plus education level, region, smoking status, SBP, DBP, urban or rural residents, physical activity (low, moderate, high) at baseline, as well as cumulative average fiber intake, and sodium to potassium intake ratio, and fat intake (% energy)

In the stratified analyses, age, sex, BMI, abdominal obesity, energy intake, total cholesterol intake, fiber intake, the percentages energy from total carbohydrate and total fat, and variety score of protein sources did not significantly modify the association between the percentage energy from total protein intake and the risk of new-onset diabetes (All *P* interactions >0.05) (Additional file 1: Table S4).

For specific protein sources, there were U-shaped associations of the percentages energy from whole grain-derived (Fig. 1B) and poultry-derived (Fig. 1C) proteins with new-onset diabetes, J-shaped associations of unprocessed (Fig. 1D) or processed (Fig. 1E) red meat-derived proteins with new-onset diabetes, a reverse J-shaped association of fish-derived protein (Fig. 1F) with new-onset diabetes, L-shaped associations of eggs-derived (Fig. 1G) and legumes-derived (Fig. 1H) proteins with new-onset diabetes, and a reverse L-shaped association of refined grain-derived protein (Fig. 1I) with new-onset diabetes (all *P* values for nonlinearity <0.001). That is, for each protein, there is a window of consumption (appropriate level) where the risk of diabetes is lower.

Similar results were found when different sources of proteins intake (% of energy) were assessed as categories. In detail, the appropriate levels (% of energy) of specific sourced proteins associated with lower risk of new-onset diabetes were 0≤0.7 (the 1–3 quartiles among consumers) for whole-grain protein, 0≤1.2 (the 1–3 quartiles among consumers) for poultry protein, 0.5≤2.1 (the 2–3 quintiles) for unprocessed red meat protein, 0≤0.8 (the 1–3 quartiles among consumers) for processed red meat protein, 0≤1.4 (the 1–3 quartiles among consumers) for fish protein, ≥0.1 (the 2–5 quintiles) for eggs protein, ≥0.4 (the 2–5 quintiles) for legumes protein, and <3.8 (the first quintile) for refined grain protein (Additional file 1: Table S5-6).

### Relation of variety score of protein sources with new-onset diabetes

**Fig. 2:**
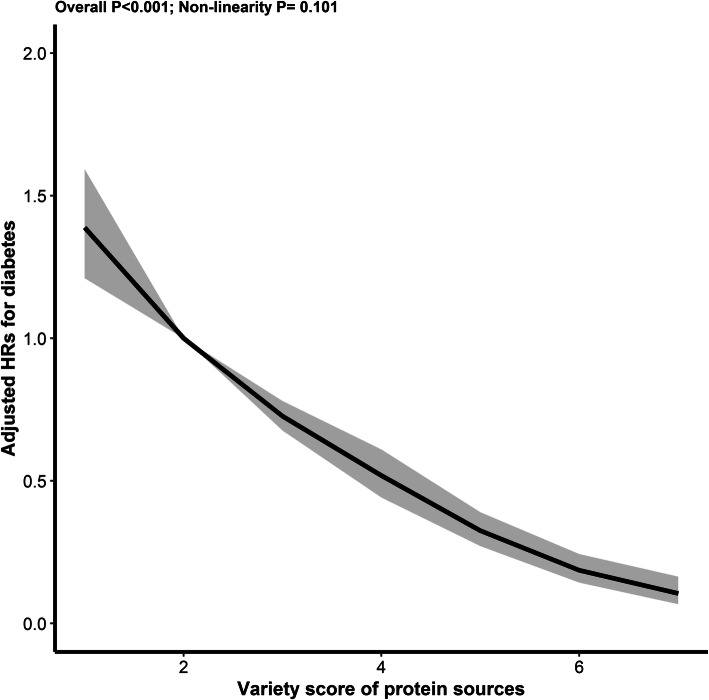
The association between variety score of protein sources and new-onset diabetes^*^. ^*^Adjusted for age, sex, BMI, occupations, education level, region, smoking status, SBP, DBP, urban or rural residents, physical activity (low, moderate, high) at baseline, as well as cumulative average total energy intake, fiber intake, sodium to potassium intake ratio, and fat intake (% energy)

Overall, there was an inverse association between the variety score of protein sources and new-onset diabetes (per score increment, HR, 0.69; 95% CI 0.65–0.72) (Fig. 2, Table 3). Accordingly, when the variety score of protein sources was assessed in quartile, compared with those in the first quartile (<2), a significantly lower risk of new-onset diabetes was found in participants in the 2–4 quartiles (≥2; HR, 0.49; 95% CI, 0.40–0.60) (Table 3). Similar results were found with the exclusion of early new-onset diabetes that occurred within the first 2 years after enrollment, using multiple imputations to handle missing values at baseline, further adjusting for dietary cholesterol intake, accounting for the competing risk of death, or after the removal of any one kind of protein from the protein variety score (Additional file 1: Table S7).

**Table 3:** The association between variety score of protein sources and new-onset diabetes

Protein variety score	No. of case	Person-years	Model 1	*P* value	Model 2	*P* value
			HR (95%CI)		HR (95%CI)	
Continuous (per score increment)	1100	158931	0.70 (0.67, 0.73)	<0.001	0.69 (0.65, 0.72)	<0.001
Quartile						
Q1 (<2)	132	11346	*Ref*		*Ref*	
Q2 (2)	245	24003	0.84 (0.67, 1.05)	0.125	0.87 (0.69, 1.09)	0.223
Q3 (3)	273	35956	0.54 (0.43, 0.67)	<0.001	0.55 (0.43, 0.69)	<0.001
Q4 (≥4)	450	87625	0.30 (0.24, 0.37)	<0.001	0.30 (0.24, 0.37)	<0.001
*P* for trend			<0.001		<0.001	
Categories						
Q1 (<2)	132	11346	*Ref*		*Ref*	
Q2–4 (≥2)	968	147584	0.45 (0.37, 0.54)	<0.001	0.49 (0.40, 0.60)	<0.001

Model 1: Adjusted for age, sex, BMI, and occupations at baseline, and cumulative average total energy intake

Model 2: Adjusted for variables in model 1 plus education level, region, smoking status, SBP, DBP, urban or rural residents, physical activity (low, moderate, high) at baseline, as well as cumulative average fiber intake, sodium to potassium intake ratio, and fat intake (% energy)

Moreover, in the stratified analyses, age, sex, BMI, abdominal obesity, energy intake, total cholesterol intake, fiber intake, and the percentages energy from total carbohydrate, total fat, and total protein intake did not significantly modify the inverse association between variety score of protein sources and new-onset diabetes (All *P*-interactions >0.05) (Additional file 1: Table S8).

## Discussion

Findings from our study suggested a U-shaped association between total protein intake and risk of new-onset diabetes in the general Chinese population. Furthermore, we extended evidence for protein from specific protein sources and found that there were U-shaped, J-shaped, reverse J-shaped, L-shaped, or reverse L-shaped associations between proteins from different food sources and new-onset diabetes. That is, when the percentages of energy from these food-derived protein intakes were relatively low, there were negative correlations or no correlations between these food-derived proteins intake and diabetes risk; however, when intake exceeded certain thresholds, the risks of new-onset diabetes increased or reached a plateau. More importantly, we first demonstrated that a greater variety of proteins with appropriate quantities from different food sources was inversely related to new-onset diabetes risk.

In Western countries, previous studies have revealed positive [31–36] or non-significant [10, 11, 37] associations between total protein intake and diabetes risks. However, our results demonstrated a U-shaped association of total protein intake and diabetes in the general Chinese population, with the lowest risk at 10.6%≤14.0% of energy from total protein. The inconsistent results might result from a different dietary pattern, including higher proportions of energy from total protein (16.3–21.6%) [31–37] in Western countries than that in China (10.9–13.5%) and different major sources of protein. In detail, animal protein (mainly derived from red meat, poultry, fish, egg, and dairy products) and plant protein (mainly derived from grains, legumes, and vegetables), respectively, account for major protein sources (about 60–70% of total protein) for Western countries and China [31–37]. Of note, recommendations from the Mediterranean diet and National Cholesterol Education Program suggested approximately 15% of energy from total protein [38, 39], which are slightly higher than our findings (10.6%≤14.0%). In fact, the recommended protein sources are mainly high-quality protein ones, including dairy products, legumes, and fish. In our study, relatively higher intakes of fish and legumes were still related to a lower risk of new-onset diabetes, which may partly explain the potential differences.

### Animal proteins (red meat, poultry, fish, and eggs) and diabetes risk

To date, only two previous studies [10, 11] had investigated the relation of specific protein sources with incident diabetes. Possibly due to the relatively limited sample size (*N*= 2322), Virtanen et al. found that none of the specific sourced protein was associated with diabetes risk in Finland men [10]. However, the Rotterdam study (*N*=6822) [11] reported that there were significantly positive relations of proteins from meat, fish, and dairy with new-onset diabetes. Consistently, we also observed that higher red meat-derived protein was associated with increased diabetes risk. Of note, the Rotterdam study only hypothesized a linear association and assessed dietary protein as per 5% of energy increment and therefore may ignore more information provided by the continuous dietary data. Our present study, with a larger sample size and the assessments of dietary protein data continuously and categorically, first reported the J-shaped associations between unprocessed or processed red meat and new-onset diabetes. Moreover, we further found that the relationship between poultry-derived protein and new-onset diabetes also followed a U-shape. Previous studies had indicated that the metabolites of meat protein, including branched-chain and aromatic amino acids can undermine the normal regulation of glucose and insulin levels by activating the mammalian target of rapamycin (mTOR) [40, 41]. Moreover, the co-content of heme iron, saturated fat, nitrites, and advanced glycation end products in animal protein might also attribute to the detrimental effects [42]. On the other hand, as one of the main daily energy suppliers, protein plays essential roles in normal physiological processes and may improve the blood glucose response [43]. The U- or J-shaped association suggested that red meat and poultry are major protein sources, and therefore, the moderate protein intake from red meat and poultry may possibly also play some roles in maintaining the normal glucose metabolism. However, further studies are required to confirm our findings.

Furthermore, we found a reverse J-shaped association of the fish-derived protein with new-onset diabetes. Proteins in fish are deemed as high-quality ones, which can lead to a greater satiety level, a slower decline in satiety and improve insulin sensitivity when compared to other animal proteins [44–46]. However, consistent with the findings in the Rotterdam Study, the reverse J-shaped association indicated that too high fish-derived protein may possibly also relate to an increased risk of diabetes [47].

More importantly, consistent with findings in European populations [48], our study showed an L-shaped association between the intake of egg-derived protein and diabetes risk. The potential benefits of optimal egg-derived protein might be due to its metabolites, such as alpha-glucosidase inhibitory peptides, ACE inhibitory peptides, DPP-4 inhibitory peptides, which showed effects on the improvement of glucose tolerance, postprandial hyperglycemia, and insulin resistance [49–51].

### Plant proteins (grain and legumes) and diabetes risk

Possibly due to the relatively lower intake level of plant protein (percentages energy from grains= 3%; percentages energy from legumes and nuts= 0.5%), the Rotterdam Study [11] reported that there were no significant relations between plant proteins from grains or legumes with diabetes risk. Our study showed that participants with higher refined, whole grain-derived proteins intake had a significantly higher risk of diabetes. That might be plausible, since amino acids from the metabolism of grain storage proteins like lysine and methionine, have been reported to be positively associated with obesity [52], a crucial risk factor for diabetes. Of note, previous studies really had found that dietary fiber in whole grain might be beneficial to glycemic control and was inversely associated with risk of diabetes [53–55]. On the one hand, our study also showed that there was a U-shaped association between whole grain-derived protein intake and new-onset diabetes. That is, optimal whole grain-derived protein intake was related to a lower risk of new-onset diabetes. On the other hand, fiber intake had been adjusted in the regression models, which means that the association between whole grain-derived protein intake and new-onset diabetes may be partly independent of the fiber intake. More importantly, our findings indicated a lower diabetes risk for those who consumed more legume-derived protein. Benefits from legumes sourced protein include reduced adiposity, and insulin secretion from pancreatic β cells, and improved insulin sensitivity [56–58]; these could benefit glucose metabolism.

### Variety of protein sources and diabetes risk

Furthermore, our present study expands the results of previously published studies by demonstrating that a greater variety of proteins with appropriate quantity from different food sources was significantly associated with the lower risk of new-onset diabetes. The possible interpretation is that consuming a variety of proteins may guarantee the intake of different essential amino acids and correlate with better nutritional status and microbiota diversity [59]. However, more studies are needed to further examine the underlying mechanisms involved in this association.

Our study is a relatively large-scale, nationally prospective cohort among the general population. It used repeated measurements of 3 days 24-h recall data to represent long-term dietary intake status and minimize within-person variation and included adjustments for a comprehensive range of covariates, and multiple sensitivity analyses and subgroup analyses to ensure the robustness of the study findings. However, there are some limitations needed to be mentioned. First, diabetes incidence may be underestimated with only self-reported diabetes through questionnaires in all rounds and fasting glucose only in the 2009 round. More frequent measurements of glucose levels would improve the accuracy of the results. Second, compared with excluded individuals, those included in the current study (Additional file 1: Table S1) had higher physically active and education levels, seemed to be older, more likely to be current smokers and drinkers, and less likely to live in urban residence. Although we have fully adjusted for these potential covariates and did not find any significant modification effects, unmeasured and residual confounding remains possible. Third, the present study could not determine causation due to the observational design. Fourth, the information about the family history of diabetes and isoflavones was unavailable in the CHNS; therefore, we could not examine whether the history of diabetes and isoflavones may affect our findings. Fifth, proteins are not consumed isolated in the diet; other nutrients accompanied with proteins may potentially influence the pathophysiology of diabetes. Although we had adjusted the dietary intake of total cholesterol, fat, and fiber in the regression models, and made a series of related stratified analyses, we could not evaluate the possible modifying effect of other unknown or unavailable nutrients from the foods. Moreover, dietary measurements in CHNS were derived from self-reported dietary 24-h recalls, which may be affected by recall bias. Nevertheless, it is one of the most common methods for dietary intake data and has been used by some precious important cohorts [19, 60, 61]. More importantly, the accuracy of 24-h dietary recall for the evaluation of energy and nutrient intake has been validated by lots of previous studies [20, 62, 63]. Overall, further studies are still needed to confirm the results.

## Conclusions

In summary, our results suggested that there was an inverse association between the variety of proteins with appropriate quantity from different food sources (whole and refined grain, processed and unprocessed red meat, poultry, fish, egg, and legumes) and new-onset diabetes in general Chinese adults. If further confirmed, these findings encouraged the consumption of a balanced diet and emphasized the particularly important role of a moderate quantity of proteins from diverse food sources for the primary prevention of diabetes.

## Supplementary Information

**Additional File 1: Fig. S1-S2. Fig. S1.** Flow chart of study participants. **Fig. S2**. The longitudinal comparisons of macronutrients intake from 1997-2011. **Table S1-S8. Table S1.** Personal characteristics of the included and excluded participants. **Table S2.** Food sources of dietary protein intake. **Table S3.** Sensitivity analysis of total protein intake (% of energy) with new-onset diabetes. **Table S4.** Stratified analyses of the association between total protein intake (% of energy) and new-onset diabetes. **Table S5.** The relations of specific sourced proteins intake (proportion of non-consumers < 20%) with new-onset diabetes. **Table S6.** The relations of specific sourced proteins intake (proportion of non-consumers ≥ 20%) with new-onset diabetes. **Table S7.** The sensitivity analysis of association between variety score of protein sources and new-onset diabetes. **Table S8.** Stratified analyses of the association between variety score of protein sources and new-onset diabetes.

## Data Availability

The datasets generated and analyzed during the current study are available the CHNS official website (http://www.cpc.unc.edu/projects/china).

## Abbreviations

CHNS: China Health and Nutrition Survey
FCTs: China food composition tables
SBP: Systolic blood pressure
DBP: Diastolic blood pressure
MET: Metabolic equivalent
SDs: Standard deviations
BMI: Body mass index
RCS: Restricted cubic splines
