# Supplementary material for: Variety and quantity of dietary protein intake from different sources and risk of new-onset diabetes: a Nationwide Cohort Study in China
Source: BMC Med. 2022 Jan 13;20:6. doi: 10.1186/s12916-021-02199-8 (PMC8756636; doi:10.1186/s12916-021-02199-8)
Supplement: Supplementary file 1 — Additional File 1: Fig. S1-S2. Fig. S1. Flow chart of study participants. Fig. S2. The longitudinal comparisons of macronutrients intake from 1997-2011. Table S1-S8. Table S1. Personal characteristics of the included and excluded participants. Table S2. Food sources of dietary protein intake. Table S3. Sensitivity analysis of total protein intake (% of energy) with new-onset diabetes. Table S4. Stratified analyses of the association between total protein intake (% of energy) and new-onset diabetes. Table S5. The relations of specific sourced proteins intake (proportion of non-consumers < 20%) with new-onset diabetes. Table S6. The relations of specific sourced proteins intake (proportion of non-consumers ≥ 20%) with new-onset diabetes. Table S7. The sensitivity analysis of association between variety score of protein sources and new-onset diabetes. Table S8. Stratified analyses of the association between variety score of protein sources and new-onset diabetes. [file 12916_2021_2199_MOESM1_ESM.doc]

94532 person-waves in CHNS data from 1997 to 2015

75466 person-waves

Excluded n=19066 person-waves

Age<18 years, n=17672

Being Pregnant, n=360

Missing diabetes diagnosis, n=1034

N=16451 participants

A cohort based on participants with two or more survey waves

N=16895 (66547 person-waves)

Excluded n= 8919 person-waves

Participants with only one survey wave

Excluded n= 444 participants

Having diabetes at baseline

N=16348 participants

Excluded n= 103 participants

Missing protein intake data

Included in the final study

N=16260 participants

Excluded n=88 participants

Outlier of energy intake (male: ≥4200 or <800 kcal/day, female: ≥3600 or <600 kcal/day)

**Fig. S1. Flow chart of study participants**

**
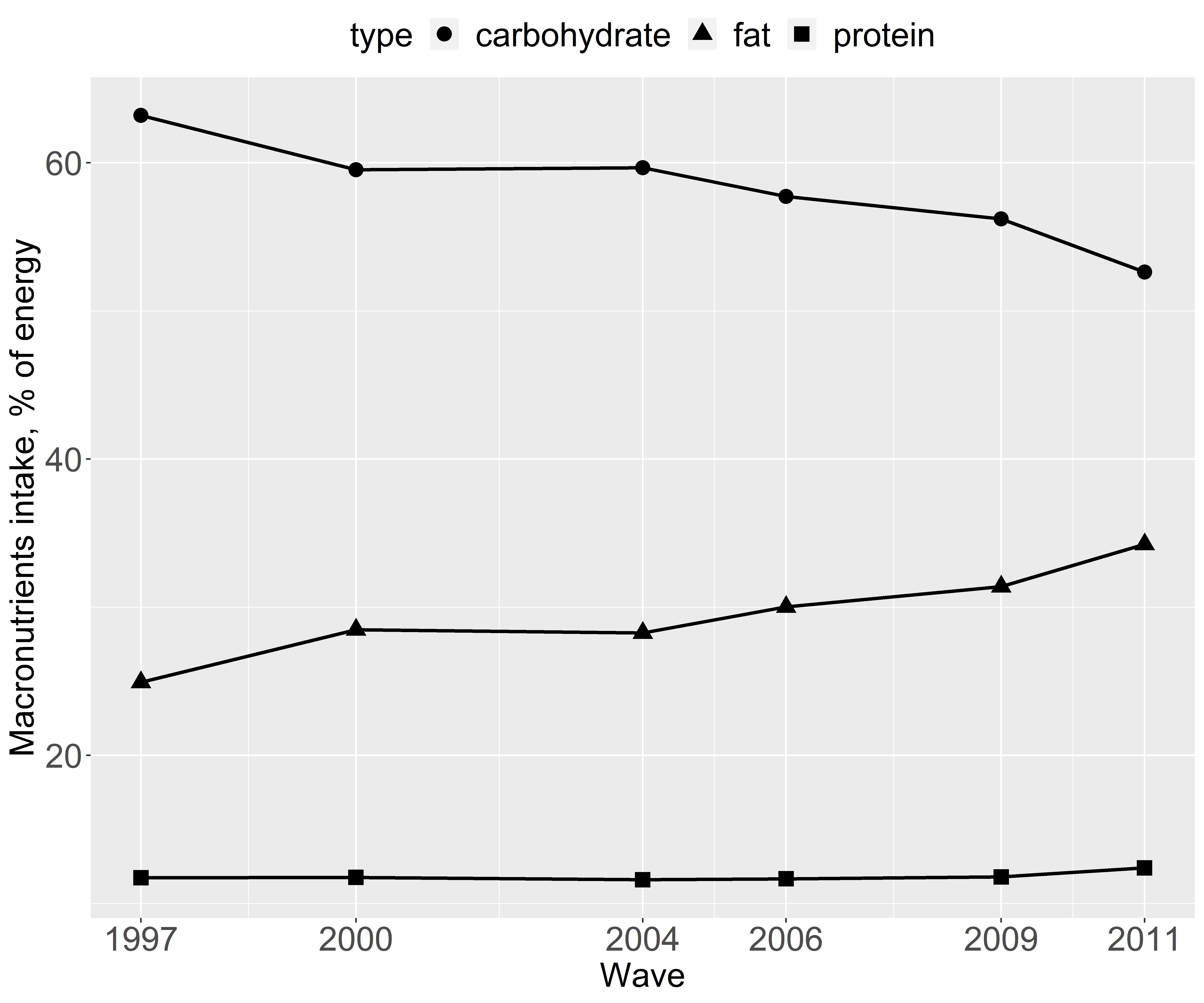
**

**Fig. S2. The longitudinal comparisons of macronutrients intake from 1997-2011**

**Table S1. Personal characteristics of the included and excluded participants.**

|  | **Included** | **Excluded^*^** |
| --- | --- | --- |
| **N** | 16895 | 9065 |
| **Age, years** | 49.2 (38.3,60.6) | 37.9 (25.2,55.5) |
| **Male, No. (%)** | 32240 (48.4) | 4608 (46.3) |
| **Systolic blood pressure, mmHg** | 120.0 (110.0,132.0) | 120.0 (110.0,130.0) |
| **Diastolic blood pressure, mmHg** | 80.0 (70.7,85.3) | 78.7 (70.0,83.3) |
| **Body mass index, kg/m^2^** | 23.0 (20.8,25.5) | 22.7 (20.4,25.4) |
| **Waist circumference, cm** | 81.0 (74.0,89.0) | 80.0 (72.0,88.0) |
| **Physical activity, MET-hours/week** | 99.6 (31.7,200.5) | 85.9 (27.8,169.3) |
| **Urban residents, No. (%)** | 21788 (32.7) | 4782 (48.0) |
| **Smoking status, No. (%)** |  |  |
| Never | 45151 (68.6) | 6886 (74.6) |
| Former | 1990 (3.0) | 197 (2.1) |
| Current | 18686 (28.4) | 2148 (23.3) |
| **Drinking status, No. (%)** |  |  |
| Never | 43823 (67.2) | 6447 (70.8) |
| No more than once a month | 2384 (3.7) | 431 (4.7) |
| Once or twice a month | 4162 (6.4) | 619 (6.8) |
| Once or twice a week | 5341 (8.2) | 659 (7.2) |
| 3-4 times a week | 2903 (4.4) | 341 (3.7) |
| Almost every day | 6632 (10.2) | 606 (6.7) |
| **Regions^†^, No. (%)** |  |  |
| North | 27197 (40.9) | 4173 (41.9) |
| South | 39350 (59.1) | 5780 (58.1) |
| **Occupation, No. (%)** |  |  |
| White-collar worker | 7211 (10.9) | 1489 (15.2) |
| Blue-collar worker | 12717 (19.3) | 2397 (24.4) |
| Farmer | 18963 (28.8) | 1461 (14.9) |
| Unemployed or other | 26974 (41.0) | 4464 (45.5) |
| **Education, No. (%)** |  |  |
| Illiteracy | 13439 (20.9) | 847 (9.0) |
| Primary school | 13137 (20.5) | 1302 (13.9) |
| Middle school | 20576 (32.0) | 3274 (34.9) |
| High school or above | 17051 (26.6) | 3950 (42.1) |
| **Dietary intake** |  |  |
| Energy, kcal/day | 2133.6 (1723.7,2592.4) | 2017.5 (1612.8,2472.6) |
| Refined grains, g/day | 373.3 (283.3,483.3) | 350.0 (258.3,450.0) |
| Whole grains, g/day | 0 (0,8.3) | 0 (0,8.3) |
| Legumes, g/day | 33.3 (0,80.0) | 33.3 (0,75.0) |
| Vegetables, g/day | 270.2 (184.7,375.5) | 246.7 (167.4,341) |
| Fruits, g/day | 0 (0,8.3) | 0 (0,23.0) |
| Nuts, g/day | 0 (0,0) | 0 (0,0) |
| Red meat, g/day | 50 (3.3,100.0) | 53.3 (16.7,100.0) |
| Poultry, g/day | 0 (0,0) | 0 (0,0) |
| Processed meat, g/day | 0 (0,0) | 0 (0,0) |
| Dairy products, g/day | 0 (0,0) | 0 (0,0) |
| Eggs, g/day | 14.7 (0,37.7) | 14.7 (0,43.5) |
| Aquatic products, g/day | 0 (0,30.4) | 0 (0,29.0) |
| Dietary variety score | 5.0 (4.0,6.0) | 5.0 (4.0,7.0) |

Values are presented as mean ± standard deviation (SD) for normally distributed continuous variables, median (interquartile range [IQR]) for non-normally distributed continuous variables, and proportions for categorical variables, respectively.

^*^Excluded from the analysis due to missing diabetes diagnosis (1034 person waves) and those with only one survey wave (8919 person waves).

**Table S2. Food sources of dietary protein intake.**

| **Protein groups** | **Subcategories (percent of total protein)** | | **Items** |
| --- | --- | --- | --- |
| Animal protein | Red meat (16%) | Unprocessed red meat | Beef, lamb or pork, and its offal, etc. |
|  |  | Processed meat | Processed meat Bacon, ham sausage, sauced beef, hotdogs, beef hotdogs, chicken or turkey hotdogs, preserved meat, etc. |
|  | Egg (5%) | | Eggs, duck eggs, goose eggs, preserved egg, salted duck egg, etc. |
|  | Poultry (3%) | | Chicken, turkey, duck, goose, chicken wings, chicken legs, chicken claws, etc. |
|  | Fish (5%) | | fishes |
|  | Dairy (1%) | | All fluid milk products and many foods made from milk that retain their calcium content, such as yogurt, cheese, skim milk, whole milk, etc. |
|  | Seafood | | shrimp, shellfish, crabs, cucumber, squid, octopus, etc. |
|  | Residual animal protein from other sources | | All other animal protein not in the groups above |
| Plant protein | Grains (45%) | Whole grain | Wheat, barley, millet, millet flour, sorghum rice, corn, maize meal, black rice, etc. |
|  |  | Refined grain | White flour, white rice, steamed bun, noodle, etc. |
|  | Legumes (11%) | | Tofu or other soy products; peas or beans, etc. |
|  | Nuts | | Peanuts, walnuts, sunflower seed, cashew nut, gingeli, almond and other nuts. |
|  | Residual plant protein from other sources | | All other plant protein not in the groups above (e.g. vegetables and fruits). |

**Table S3. Sensitivity analysis of total protein intake (% of energy) with new-onset diabetes**

| Analysis | Analysis 1 | | Analysis 2 | | Analysis 3 | | Analysis 4 | |
| --- | --- | --- | --- | --- | --- | --- | --- | --- |
| Protein,  % of energy | Model 1 | *P* value | Model 1 | *P* value | Model 1 | *P* value | Model 1 | *P* value |
|  | HR (95%CI) |  | HR (95%CI) |  | HR (95%CI) |  | HR (95%CI) |  |
| Q1(<10.6) | 1.17(0.94, 1.45) | 0.166 | 1.13(0.93,1.36) | 0.211 | 1.17(0.93, 1.46) | 0.181 | 1.17 (0.95, 1.44) | 0.130 |
| Q2(10.6-< 11.6) | ref |  | ref |  | ref |  | ref |  |
| Q3(11.6-<12.6) | 0.96(0.78, 1.20) | 0.744 | 0.94(0.78,1.13) | 0.488 | 0.90(0.72, 1.12) | 0.342 | 0.90 (0.73, 1.10) | 0.290 |
| Q4(12.6-<14.0) | 0.94(0.75, 1.18) | 0.610 | 0.95(0.78,1.17) | 0.654 | 0.96(0.76, 1.20) | 0.697 | 0.90 (0.73, 1.11) | 0.310 |
| Q5(≥14.0) | 1.29(1.02, 1.64) | 0.037 | 1.3(1.05,1.62) | 0.019 | 1.21(0.95, 1.54) | 0.125 | 1.29(1.04, 1.61) | 0.023 |
| Categories |  |  |  |  |  |  |  |  |
| Q1(<10.6) | 1.20(1.00, 1.44) | 0.055 | 1.19(1.01,1.40) | 0.033 | 1.22(1.01, 1.49) | 0.041 | 1.25 (1.05, 1.50) | 0.011 |
| Q2-4(10.6-<14.0) | ref |  | ref |  | ref |  | ref |  |
| Q5(≥14.0) | 1.33(1.10, 1.62) | 0.004 | 1.35(1.14,1.60) | <0.001 | 1.28(1.05, 1.56) | 0.015 | 1.40 (1.17, 1.67) | <0.001 |

Model 1: Adjusted for age, sex, BMI, occupations, education level, region, smoking status, SBP, DBP, urban or rural residents, physical activity (low, moderate, high) at baseline, as well as cumulative average total energy intake, fiber intake, sodium to potassium intake ratio, and fat intake (% energy).

Analysis 1: Excluding early new-onset diabetes that occurred within the first two years after enrollment;

Analysis 2: Using multiple imputations to handle missing values at baseline;

Analysis 3: Further adjustment for dietary cholesterol;

Analysis 4: Using Fine-Gray competing risk model based on Model 1.

**Table S4. Stratified analyses of the association between total protein intake (% of energy) and new-onset diabetes ^*^**

| Sub-groups | Total protein, % of energy | | | *P* for interaction |
| --- | --- | --- | --- | --- |
|  | Q1 | Q2-4 | Q5 |  |
| Age, yrs | | |  | 0.193 |
| <60 | 1.33(1.10,1.61) | *ref* | 1.30(1.05,1.59) |  |
| ≥60 | 1.13(0.79,1.62) | *ref* | 1.67(1.23,2.27) |  |
| Sex | |  |  | 0.946 |
| Men | 1.31(1.02,1.68) | *ref* | 1.38(1.09,1.75) |  |
| Women | 1.25(1.00,1.56) | *ref* | 1.41(1.10,1.80) |  |
| BMI, kg/m^2^ |  |  |  | 0.531 |
| <24 | 1.13(0.89,1.43) | *ref* | 1.39(1.05,1.84) |  |
| ≥24 | 1.36(1.08,1.72) | *ref* | 1.42(1.15,1.76) |  |
| Abdominal obesity ^§^ | |  |  | 0.441 |
| no | 1.25(0.99,1.59) | *ref* | 1.55(1.19,2.02) |  |
| yes | 1.31(1.04,1.66) | *ref* | 1.28(1.02,1.60) |  |
| Energy, Kcal |  |  |  | 0.122 |
| <2118 (median) | 1.53(1.20,1.96) | *ref* | 1.55(1.23,1.95) |  |
| ≥2118 | 1.15(0.92,1.43) | *ref* | 1.20(0.93,1.54) |  |
| Total carbohydrate intake, % of energy | |  |  | 0.922 |
| <56 (median) | 1.30(0.98,1.73) | *ref* | 1.41(1.14,1.75) |  |
| ≥56 | 1.23(1.00,1.52) | *ref* | 1.46(1.08,1.98) |  |
| Total fat intake, % of energy | |  |  | 0.446 |
| <31 (median) | 1.20(0.97,1.50) | *ref* | 1.21(0.93,1.59) |  |
| ≥31 | 1.32(1.02,1.70) | *ref* | 1.51(1.21,1.88) |  |
| Fiber, g/d |  |  |  | 0.063 |
| <10 (median) | 1.54(1.22,1.94) | *ref* | 1.46(1.14,1.87) |  |
| ≥10 | 1.04(0.81,1.32) | *ref* | 1.35(1.07,1.71) |  |
| Total cholesterol, mg/d |  |  |  | 0.459 |
| <285 | 1.18(0.93,1.51) | *ref* | 1.52(1.11,2.09) |  |
| ≥285 | 1.25(0.92,1.69) | *ref* | 1.22(0.96,1.55) |  |
| Variety score of protein sources |  |  |  | 0.147 |
| <2 | 0.94(0.62,1.43) | *ref* | 1.96(1.10,3.49) |  |
| ≥2 | 1.26(1.05,1.52) | *ref* | 1.39(1.16,1.66) |  |

^*^ If not stratified, adjusted for age, sex, BMI, occupations, education level, region, smoking status, SBP, DBP, urban or rural residents, physical activity (low, moderate, high) at baseline, as well as cumulative average total energy intake, fiber intake, sodium to potassium intake ratio, and fat intake (% energy).

^§^ Abdominal obesity was defined as waist circumference ≥80 in women, or ≥ 90 cm in men.

**Table S5. The relations of specific sourced protein intake (proportion of non-consumers < 20%) with new-onset diabetes**

|  | **Protein intake, % of energy (divided according to quintiles)** | | | | | ***P* for trend** |
| --- | --- | --- | --- | --- | --- | --- |
|  | **Quintile 1** | **Quintile 2** | **Quintile 3** | **Quintile 4** | **Quintile 5** |  |
| ***Protein from animal food sources*** | | | | | | |
| **Unprocessed red meat-derived protein** | <0.5 | 0.5-<1.3 | 1.3-<2.1 | 2.1-<3.1 | ≥3.1 | - |
| No. of case | 279 | 211 | 203 | 216 | 191 |  |
| Person-years | 32748 | 35024 | 33243 | 31839 | 26076 | - |
| Adjusted Model 1 | Ref | 0.58(0.47, 0.70) | 0.57(0.47, 0.70) | 0.64(0.53, 0.79) | 0.83(0.67, 1.02) | 0.171 |
| Adjusted Model 2 | Ref | 0.67(0.54, 0.82) | 0.73(0.59, 0.92) | 0.91(0.71, 1.16) | 1.26(0.97, 1.64) | 0.016 |
| Adjusted Model 3 | Ref | 0.68(0.55, 0.84) | 0.76(0.61, 0.96) | 0.95(0.74, 1.21) | 1.34(1.02, 1.76) | 0.005 |
| **Egg-derived protein** | <0.1 | 0.1-<0.4 | 0.4-<0.6 | 0.6-<1.0 | ≥1.0 | - |
| No. of case | 250 | 210 | 214 | 214 | 212 |  |
| Person-years | 29483 | 37544 | 35042 | 32222 | 24638 | - |
| Adjusted Model 1 | Ref | 0.57(0.47, 0.69) | 0.57(0.47, 0.69) | 0.59(0.49, 0.72) | 0.68(0.56, 0.83) | 0.002 |
| Adjusted Model 2 | Ref | 0.57(0.47, 0.70) | 0.54(0.44, 0.67) | 0.53(0.43, 0.66) | 0.65(0.52, 0.81) | <0.001 |
| Adjusted Model 3 | Ref | 0.58(0.48, 0.71) | 0.55(0.45, 0.67) | 0.54(0.44, 0.67) | 0.67(0.54, 0.83) | <0.001 |
| ***Protein from plant food sources*** | |  |  |  |  |  |
| **Refined grain-derived protein** | <3.8 | 3.8-<4.7 | 4.7-<5.4 | 5.4-<6.4 | ≥6.4 | - |
| No. of case | 154 | 177 | 217 | 245 | 307 | - |
| Person-years | 21056 | 31434 | 34862 | 36238 | 35341 | - |
| Adjusted Model 1 | Ref | 0.79(0.63, 0.99) | 1.03(0.83, 1.28) | 1.25(1.01, 1.56) | 1.83(1.47, 2.27) | <0.001 |
| Adjusted Model 2 | Ref | 0.81(0.64, 1.03) | 1.03(0.80, 1.32) | 1.21(0.92, 1.58) | 1.76(1.29, 2.41) | <0.001 |
| Adjusted Model 3 | Ref | 0.93(0.73, 1.19) | 1.24(0.95, 1.62) | 1.56(1.15, 2.11) | 2.44(1.71, 3.50) | <0.001 |
| **Legumes-derived protein** | <0.4 | 0.4-<0.8 | 0.8-<1.3 | 1.3-<2.1 | ≥2.1 | - |
| No. of case | 218 | 219 | 205 | 222 | 236 | - |
| Person-years | 25760 | 32668 | 34016 | 34746 | 31741 | - |
| Adjusted Model 1 | Ref | 0.69(0.56, 0.84) | 0.65(0.53, 0.79) | 0.57(0.46, 0.69) | 0.63(0.52, 0.77) | <0.001 |
| Adjusted Model 2 | Ref | 0.69(0.57, 0.85) | 0.62(0.51, 0.77) | 0.57(0.46, 0.70) | 0.64(0.53, 0.79) | <0.001 |
| Adjusted Model 3 | Ref | 0.72(0.59, 0.89) | 0.68(0.55, 0.83) | 0.65(0.52, 0.80) | 0.79(0.64, 0.98) | 0.032 |

For each protein whose proportion of non-consumers was less than 20%, participants were divided into ten five groups according to quintiles of the protein intake, and the first quintile was used as the reference.

Model 1: Adjusted for age, sex, BMI and occupations at baseline, and cumulative average total energy intake.

Model 2: Adjusted for variables in model 1 plus education level, region, smoking status, SBP, DBP, urban or rural residents, physical activity (low, moderate, high) at baseline, as well as cumulative average fiber intake, sodium to potassium intake ratio, and fat intake (% energy).

Model 3: Besides all the variables in model 2, for protein from animal food sources, energy from plant-derived protein was adjusted, in addition, energy from specific sourced animal protein were further mutually adjusted; For protein from plant food sources, energy from animal-derived protein was adjusted, in addition, energy from specific sourced plant protein were further mutually adjusted.

**Table S6. The relations of specific sourced protein intake (proportion of non-consumers ≥ 20%) with new-onset diabetes**

|  | **Non-consumers** | **Protein intake, % of energy (divided according to quartiles)** | | | | ***P* for trend** |
| --- | --- | --- | --- | --- | --- | --- |
|  |  | **Quartile 1** | **Quartile 2** | **Quartile 3** | **Quartile 4** |  |
| ***Protein from animal food sources*** | | | | | | |
| **Processed red meat-derived protein** | - | <0.2 | 0.2-<0.4 | 0.4-<0.8 | ≥0.8 | - |
| No. of case | 935 | 28 | 50 | 37 | 50 |  |
| Person-years | 121587 | 12113 | 10574 | 8732 | 5926 | - |
| Adjusted Model 1 | Ref | 0.29(0.19, 0.43) | 0.58(0.43, 0.79) | 0.46(0.32, 0.65) | 1.10(0.82, 1.48) | <0.001 |
| Adjusted Model 2 | Ref | 0.32(0.21, 0.48) | 0.63(0.46, 0.86) | 0.51(0.36, 0.72) | 1.20(0.89, 1.62) | 0.002 |
| Adjusted Model 3 | Ref | 0.32(0.21, 0.48) | 0.64(0.46, 0.87) | 0.51(0.36, 0.72) | 1.22(0.91, 1.65) | 0.003 |
| **Poultry-derived protein** | - | <0.3 | 0.3-<0.7 | 0.7-<1.2 | ≥1.2 | - |
| No. of case | 708 | 89 | 108 | 102 | 93 |  |
| Person-years | 81222 | 25025 | 21115 | 18213 | 13355 | - |
| Adjusted Model 1 | Ref | 0.39(0.31, 0.49) | 0.56(0.45, 0.69) | 0.63(0.50, 0.78) | 0.81(0.65, 1.03) | <0.001 |
| Adjusted Model 2 | Ref | 0.40(0.31, 0.51) | 0.58(0.46, 0.72) | 0.63(0.50, 0.80) | 0.89(0.70, 1.13) | <0.001 |
| Adjusted Model 3 | Ref | 0.40(0.31, 0.51) | 0.57(0.46, 0.72) | 0.63(0.50, 0.79) | 0.87(0.68, 1.11) | <0.001 |
| **Fish-derived protein** | - | <0.4 | 0.4-<0.8 | 0.8-<1.4 | ≥1.4 | - |
| No. of case | 524 | 156 | 139 | 146 | 135 |  |
| Person-years | 59302 | 29562 | 26219 | 24515 | 19333 | - |
| Adjusted Model 1 | Ref | 0.52(0.43, 0.63) | 0.57(0.47, 0.70) | 0.60(0.49, 0.73) | 0.68(0.56, 0.82) | <0.001 |
| Adjusted Model 2 | Ref | 0.54(0.44, 0.66) | 0.60(0.49, 0.74) | 0.64(0.52, 0.78) | 0.72(0.59, 0.89) | <0.001 |
| Adjusted Model 3 | Ref | 0.52(0.43, 0.64) | 0.58(0.47, 0.71) | 0.61(0.50, 0.76) | 0.70(0.57, 0.87) | <0.001 |
| ***Protein from plant food sources*** | |  |  |  |  |  |
| **Whole grain-derived protein** | - | <0.1 | 0.1-<0.4 | 0.4-<0.7 | ≥0.7 | - |
| No. of case | 622 | 89 | 93 | 123 | 173 |  |
| Person-years | 85603 | 20198 | 18145 | 17436 | 17548 | - |
| Adjusted Model 1 | Ref | 0.48(0.38, 0.61) | 0.58(0.46, 0.73) | 0.76(0.62, 0.93) | 1.10(0.92, 1.32) | 0.336 |
| Adjusted Model 2 | Ref | 0.48(0.38, 0.62) | 0.53(0.42, 0.68) | 0.62(0.50, 0.77) | 0.91(0.75, 1.11) | 0.002 |
| Adjusted Model 3 | Ref | 0.46(0.36, 0.59) | 0.52(0.41, 0.66) | 0.60(0.48, 0.75) | 0.99(0.81, 1.22) | 0.005 |

For each protein whose proportion of non-consumers was over 20%, consumers were divided into four groups according to quartiles and non-consumers were used as the reference.

Model 1: Adjusted for age, sex, BMI and occupations at baseline, and cumulative average total energy intake.

Model 2: Adjusted for variables in model 1 plus education level, region, smoking status, SBP, DBP, urban or rural residents, physical activity (low, moderate, high) at baseline, as well as cumulative average fiber intake, sodium to potassium intake ratio, and fat intake (% energy).

Model 3: Besides all the variables in model 2, for protein from animal food sources, energy from plant-derived protein was adjusted, in addition, energy from specific sourced animal protein were further mutually adjusted; For protein from plant food sources, energy from animal-derived protein was adjusted, in addition, energy from specific sourced plant protein were further mutually adjusted.

**Table S7. The sensitivity analysis of association between variety score of protein sources and new-onset diabetes**

| Protein variety score (Per score increment) | Model 1 | *P* value |
| --- | --- | --- |
|  | HR (95%CI) |  |
| Analysis 1 | 0.71(0.68, 0.75) | <0.001 |
| Analysis 2 | 0.70(0.67,0.73) | <0.001 |
| Analysis 3 | 0.72(0.68, 0.76) | <0.001 |
| Analysis 4 | 0.71(0.68, 0.75) | <0.001 |
| Analysis 5: |  |  |
| Excluding unprocessed red meat protein | 0.68(0.64, 0.71) | <0.001 |
| Excluding refined grain protein | 0.69(0.66, 0.72) | <0.001 |
| Excluding legume protein | 0.68(0.64, 0.71) | <0.001 |
| Excluding egg protein | 0.68(0.65, 0.72) | <0.001 |
| Excluding processed protein | 0.69(0.66, 0.73) | <0.001 |
| Excluding poultry protein | 0.67(0.64, 0.71) | <0.001 |
| Excluding fish protein | 0.65(0.62, 0.69) | <0.001 |
| Excluding whole grain protein | 0.69(0.65, 0.72) | <0.001 |

Model 1: Adjusted for age, sex, BMI, occupations, education level, region, smoking status, SBP, DBP, urban or rural residents, physical activity (low, moderate, high) at baseline, as well as cumulative total energy intake, fiber intake, sodium to potassium intake ratio, and fat intake (% energy).

Analysis 1: Excluding early new-onset diabetes that occurred within the first two years after enrollment;

Analysis 2: Using multiple imputations to handle missing values at baseline;

Analysis 3: Further adjustment for dietary cholesterol;

Analysis 4: Using Fine-Gray competing risk model based on Model 1;

Analysis 5: Removal of any one kind of protein from the protein variety score.

**Table S8. Stratified analyses of the association between variety score of protein sources and new-onset diabetes ^*^**

| Sub-groups | Variety score of protein | *P* for interaction |
| --- | --- | --- |
|  | Per score increment |  |
| Age, yrs |  | 0.428 |
| <60 | 0.70(0.66,0.74) |  |
| ≥60 | 0.67(0.61,0.74) |  |
| Sex |  | 0.472 |
| Men | 0.70(0.65,0.75) |  |
| Women | 0.68(0.63,0.72) |  |
| BMI, kg/m^2^ |  | 0.977 |
| <24 | 0.68(0.63,0.73) |  |
| ≥24 | 0.68(0.64,0.73) |  |
| Abdominal obesity ^§^ |  | 0.419 |
| no | 0.70(0.65,0.75) |  |
| yes | 0.67(0.63,0.72) |  |
| Energy, Kcal |  | 0.273 |
| <2121 (median) | 0.67(0.63,0.72) |  |
| ≥2121 | 0.71(0.66,0.75) |  |
| Total carbohydrate intake, % of energy |  | 0.614 |
| <56 (median) | 0.70(0.65,0.75) |  |
| ≥56 | 0.68(0.64,0.73) |  |
| Total fat intake, % of energy |  | 0.907 |
| <31 (median) | 0.69(0.64,0.73) |  |
| ≥31 | 0.69(0.65,0.74) |  |
| Fiber, g/d |  | 0.303 |
| <10 (median) | 0.67(0.63,0.72) |  |
| ≥10 | 0.70(0.66,0.75) |  |
| Total cholesterol, mg/d |  | 0.190 |
| <285 | 0.74(0.69,0.80) |  |
| ≥285 | 0.69(0.64,0.75) |  |
| Total protein intake, % of energy |  | 0.485 |
| <10.6 | 0.72(0.65,0.80) |  |
| 10.6-<14.0 | 0.68(0.64,0.72) |  |
| ≥14.0 | 0.67(0.60,0.74) |  |

^*^ If not stratified, adjusted for age, sex, BMI, occupations, education level, region, smoking status, SBP, DBP, urban or rural residents, physical activity (low, moderate, high) at baseline, as well as cumulative total energy intake, fiber intake, sodium to potassium intake ratio, and fat intake (% energy).

^§^ Abdominal obesity was defined as waist circumference ≥80 in women, or ≥ 90 cm in men.
